# Clinical Applications of Autophagy Proteins in Cancer: From Potential Targets to Biomarkers

**DOI:** 10.3390/ijms18071496

**Published:** 2017-07-11

**Authors:** Svetlana Bortnik, Sharon M. Gorski

**Affiliations:** 1 Canada’s Michael Smith Genome Sciences Centre, BC Cancer Agency, Vancouver, BC V5Z 1L3, Canada; sbortnik@bcgsc.ca; 2 Interdisciplinary Oncology Program, University of British Columbia, Vancouver, BC V5Z 1L3, Canada; 3 Department of Molecular Biology and Biochemistry, Simon Fraser University, Burnaby, BC V5A 1S6, Canada; 4 Centre for Cell Biology, Development, and Disease, Simon Fraser University, Burnaby, BC V5A 1S6, Canada

**Keywords:** autophagy, cancer, biomarkers, immunohistochemistry

## Abstract

Autophagy, a lysosome-mediated intracellular degradation and recycling pathway, plays multiple context-dependent roles in tumorigenesis and treatment resistance. Encouraging results from various preclinical studies have led to the initiation of numerous clinical trials with the intention of targeting autophagy in various cancers. Accumulating knowledge of the particular mechanisms and players involved in different steps of autophagy regulation led to the ongoing discovery of small molecule inhibitors designed to disrupt this highly orchestrated process. However, the development of validated autophagy-related biomarkers, essential for rational selection of patients entering clinical trials involving autophagy inhibitors, is lagging behind. One possible source of biomarkers for this purpose is the autophagy machinery itself. In this review, we address the recent trends, challenges and advances in the assessment of the biomarker potential of clinically relevant autophagy proteins in human cancers.

## 1. Introduction

Macroautophagy (herein referred to as autophagy) is a lysosome-mediated degradation and recycling process that functions in both tumor suppression and tumor progression, depending on the stage of tumorigenesis. In advanced malignancies, autophagy promotes cancer cell survival and contributes to cancer progression and drug resistance [1], hence becoming a promising target for anticancer therapy.

Numerous clinical trials investigating the pharmacologic autophagy inhibitors chloroquine (CQ) and hydroxychloroquine (HCQ) in various cancers are underway [2]. These lysosome-targeting inhibitors were previously approved by the Food and Drug Administration (FDA) for the treatment of malaria and later were repurposed for autophagy inhibition [3]. The first published results [4,5,6] demonstrate only a moderate effectiveness of HCQ in combination with chemotherapy, indicating the need for the development of more potent and selective autophagy inhibitors as well as reliable criteria for better selection of patients entering clinical trials. While autophagy inhibitors that target specific proteins involved in different steps of the autophagy process are under development [7,8,9,10,11,12,13,14], it is crucial to determine cancer types suitable for each of these autophagy inhibition strategies. Therefore, the search for markers of autophagy, or response to autophagy modulation, in cancer patients is a research area of relevance to both clinicians and scientists.

Due to the dynamic nature of autophagy and complexity of its regulation, simultaneous assessment of several autophagy markers will likely be required to assess autophagy status. However, many autophagy proteins have been shown to function in other cellular processes, including cell survival and apoptosis, modulation of cellular trafficking, protein secretion, cell signaling, transcription, translation and membrane reorganization [15]. Each protein known to be involved in the autophagy machinery, when evaluated separately from other autophagy proteins, could have an independent biomarker potential in cancer patients, which is not necessarily related to autophagy. Caution has to be taken, therefore, when interpreting the results from studies that report association of tumoral expression of autophagy-related proteins with clinicopathological characteristics and/or patient outcomes. Two distinct goals—evaluation of autophagy status versus assessment of biomarker potential—need to be clearly separated.

Biomarkers are typically classified as “prognostic” or “predictive”. A prognostic biomarker provides information on the likely outcome of the disease in an untreated individual and is helpful in identifying patients for adjuvant systemic therapies. A predictive biomarker helps select patients who will likely benefit from a given treatment [16,17,18]. In practice, the distinction between these categories is not straightforward, and many biomarkers have both prognostic and predictive values, and also serve as therapeutic targets [17,19]. Following the definitions above, all the markers reviewed here (Table 1) fall currently into the category of “prognostic” markers, and future studies are required to establish whether they may also have predictive values for specific treatment strategies.

Numerous technologies are used to analyze biomarker values of different types of biomolecules such as DNA, RNA, proteins, and peptides [20,21]. Here, we will focus on the autophagy-related protein biomarkers, although validated autophagy-related protein biomarkers and protocols have not yet been approved for use in a clinical setting. Indeed, scoring systems for the evaluation of autophagy-related protein markers in tissues can differ dramatically between researchers. While manual scoring appears to be prevalent in the literature, some authors [22] take an automated approach, especially for large-scale studies. While acknowledging the variability of staining techniques and antibodies used, a lack of a uniform scoring system, as well as context-dependent roles of autophagy in cancers, below we summarize the recent trends in evaluating the biomarker potential of key autophagy-related proteins in various human cancers. We first provide a brief overview of the autophagy machinery itself to place the various protein biomarkers described into the context of the autophagy pathway.

## 2. Autophagy Machinery

Autophagy is a tightly-regulated multi-step process that involves more than 30 core autophagy-related (ATG) proteins [23,24]. The ATG proteins act to initiate, promote and complete the formation of double-membrane autophagosomes that fuse with lysosomes to form autolysosomes where the contents are degraded and recycled by the cell. A detailed overview of autophagic machinery has been described in several excellent reviews [1,25,26], and we include only a brief summary here.

**Figure 1:**
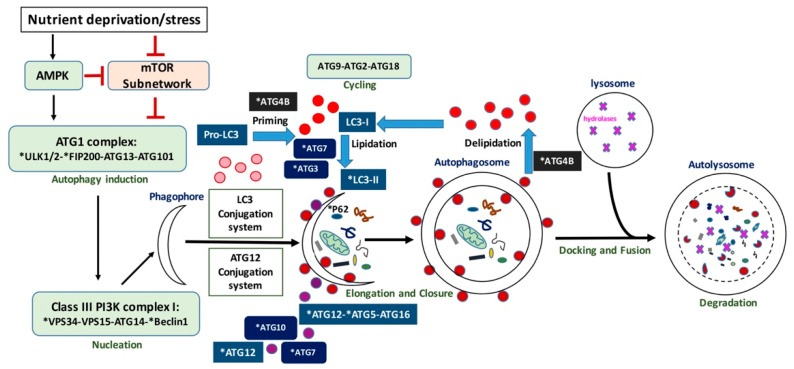
Schematic representation of autophagy. Autophagy proteins indicated with an asterisk have been investigated as potential biomarkers.

One of the main autophagy regulators is the mechanistic target of rapamycin (mTOR) protein kinase [27]; however, mTOR-independent autophagy [28] has also been described. As shown in Figure 1, autophagy is initiated by the activation of the serine/threonine Unc-51-like kinases 1 and 2 (ULK1 and ULK2), that receive signals from the master nutrient sensors mTOR and AMP-activated protein kinase (AMPK) [29]. ULK1/2 then forms a complex with ATG13, ATG101, and FIP200 [30]. This complex regulates the induction of autophagosome formation. The activity of the ULK1 kinase is required for the recruitment of the phosphatidylinositol 3-kinase catalytic subunit type 3 (PIK3C3/VPS34) to the phagophore—the cup-shaped double-membrane precursor of the autophagosome. Along with VPS15 (the PI3K regulatory subunit 4), ATG14, and the scaffold protein Beclin 1, VPS34 forms the class III PI3K complex I, which produces phosphatidylinositol 3-phosphate (PI(3)P) at the sites of phagophore nucleation. When clustered, PI(3)Ps create a cytosol-facing platform for the binding of proteins (such as WIPI/II) required for the recruitment of machineries, involved in the so-called “elongation reaction” [30,31]. This next stage in autophagosome formation requires two ubiquitin-like conjugation systems: the conjugation of ATG12 to ATG5 and ATG16L1 and, downstream to it, conjugation of Atg8 (in yeast)/microtubule-associated protein 1 light chain 3 (MAP1LC3, or LC3, in mammals) to phosphatidylethanolamine (PE). The E1-like enzyme ATG7 is involved in both conjugation processes; E2-like enzymes ATG10 and ATG3 function in ATG12-ATG5-ATG16L1 and LC3-PE conjugation systems, respectively [25,30,32,33]. In addition, the ATG12-ATG5 conjugate may act as an E3-like enzyme for the conjugation of LC3 to PE and, together with ATG16L1, is responsible for the recruitment of LC3-PE to the phagophore [34,35,36]. Prior to conjugation, LC3 is processed by the autophagy related 4B cysteine peptidase (ATG4B). ATG4B is responsible for the cleavage of the carboxyl terminus of newly synthesized pro-LC3 to provide LC3-I [37], a reaction essential for further LC3-I conjugation to PE to form a membrane-bound LC3-II during autophagosome formation. That is required for elongation of the autophagosome. ATG4B also functions in de-conjugation of LC3 from the autophagosome membrane to ensure recycling of LC3 in the cell [38]. ATG9 and its cycling system (ATG2, ATG9 and ATG18) play a role in lipid delivery to the expanding autophagosome membrane [25,30,32]. LC3 further interacts with various adaptor proteins such as p62/Sequestosome 1 (SQSTM1) that function to recruit cargo from the cytoplasm.

The process of phagophore closure is poorly understood and possibly regulated by ATG2A and ATG2B which are recruited to early autophagosomal membranes enriched in PI(3)P, where they associate with WIPI1 (WD-repeat protein interacting with phosphoinositides 1) [39]. GABA Type A Receptor-Associated Protein Like 2 (GABARAPL2/GATE-16), one of the LC3/GABARAP family members, has also been shown to mediate closure of the vesicle [35]. After closure, autophagosomes fuse with lysosomes to form autolysosomes, where the contents are degraded by hydrolases. The degradation products (e.g., amino acids) are released to the cytosol in a process involving soluble NSF-attachment protein receptors (SNAREs) and recycled by the cell [2,40].

Some mammalian orthologs of yeast ATG genes are represented by families of paralogous genes. For instance, mentioned above ATG4B belongs to the ATG4 family of cysteine proteases, which in mammals also includes ATG4A, ATG4C, and ATG4D proteins [41]. MAP1LC3 is a member of the ATG8 family, represented by three subfamilies in mammals: LC3 (LC3A, LC3B, LC3B2, LC3C), GABARAP (GABARAP, GABARAPL1), and GATE-16/GABARAPL2 [42]. Each family member, while demonstrating some functional redundancy with other members, plays distinct roles in the autophagy process [35,43]. For example, LC3s are required for the elongation step [35,44], whereas the proteins from GABARAP/GATE-16 subfamilies are thought to play important roles in autophagy initiation [44], as well as autophagosome closure [35]. A recent study by Nguyen et al. [42] provided deeper insights into the functions of different LC3/GABARAP family proteins during PINK1/Parkin mitophagy and starvation. They showed that, while these proteins are crucial for autophagosome–lysosome fusion and are likely to be important for regulating autophagosome size, they are dispensable for autophagosome formation. In spite of these new findings, LC3 remains a prominent autophagy protein and one of the main autophagosomal markers, used in a number of standard autophagy assays [45].

## 3. Candidate Autophagy-Related Biomarkers

In this review, we focus on the potential targets for autophagy modulation in cancer patients and summarize the recent reports on biomarker potential of these targets in different cancers (Table 1). Currently, the development of drugs for autophagy modulation follows two major directions: (1) lysosomal inhibitors and their derivatives; and (2) small molecule inhibitors targeting autophagy proteins. Examples of the first approach include CQ and HCQ, the main players in today’s clinical trials, as well a novel dimeric derivative of chloroquine Lys05 [46,47] which is currently being optimized for clinical trials [2]. Common autophagy markers, such as LC3B and p62, utilized in a variety of standard assays to measure autophagy turnover (also known as flux) in vitro and in vivo, may serve as useful readouts for the effects of lysosomal inhibitors on autophagy status in tumors. The potential targets from the second group include Beclin-1, ULK1, ATG4, ATG7, and VPS34.

### 3.1. LC3B

One of the best studied autophagy-related proteins, the microtubule-associated protein 1 light chain 3B (MAP1LC3B, or LC3B), has long served as an autophagy marker in multiple in vitro assays. The expression levels of LC3B protein have also been examined by immunohistochemistry (IHC) in many cancers. However, only a few recent studies (see below) differentiated among the various forms of LC3B, particularly the cytosolic (LC3B-I) and membrane-bound (LC3B-II, or LC3B “puncta”) forms [87,88].

Taking into consideration that treatment with lysosomal inhibitors results in accumulation of LC3B-II due to blockade of autophagosome-lysosome fusion, which leads to inhibition of LC3B degradation, establishing the biomarker potential of LC3B in different cancers might be useful for the selection of patients for treatment with CQ and its derivatives.

Recent systematic review and analysis by He et al. [48] showed that high protein expression of LC3B predicted adverse overall survival in breast cancer (HR = 1.98, 95% CI = 1.25–3.13), however, none of the studies included in this meta-analysis specified the pattern of LC3B staining in tumor samples. Lazova et al. [22] specifically addressed detection of LC3B puncta, rather than diffuse staining, in specimens from a relatively large cohort of breast cancer patients (*n* = 640). In this study, increased LC3B puncta expression also significantly correlated with poor prognosis. As breast cancer is a highly heterogeneous group of diseases, several authors attempted to look at LC3B expression depending on breast cancer subtype. Choi et al. [89] reported the highest LC3B expression in triple-negative breast cancers, and Zhao et al. [49] observed the association of high LC3B expression with poor overall survival and disease progression in patients with this cancer subtype. In these two studies the staining pattern (puncta vs. diffuse) was not specified. In the study by Lazova et al. [22], LC3B puncta expression was highest in the HER2/neu-positive subtype, followed by the triple-negative, luminal B, and luminal A subtypes. Interestingly, association of LC3B expression with poor outcomes reached significance only in luminal A tumors; the prognostic associations in the other 3 subtypes (luminal B, HER2/neu positive, and triple negative) did not reach statistical significance, possibly due to the limited number of patient samples [22]. Another study by Cha et al. [90] compared expression of LC3B along with other autophagy-related proteins, including Beclin-1 and p62, in invasive lobular carcinoma (ILC) versus invasive ductal carcinoma (IDC), and found that the expression of all these proteins was significantly higher in IDC. These results indicate a clear need for additional studies in large patient cohorts to determine LC3B prognostic values in different breast cancer histological and molecular subtypes.

The association of high LC3B expression with aggressive disease and poor outcomes was repeatedly reported in other cancer types, including gastric adenocarcinoma [50,51,52], colorectal cancers [53,54,55,56], melanoma [22], astrocytoma [57], esophageal cancer [58], oral squamous cell carcinoma [59] and hepatocellular carcinoma [60]. Interestingly, high expression of LC3B was associated with decreased overall survival in the KRAS-mutated subgroup of colorectal cancers, but not in the KRAS-wildtype [53]. This finding together with the previous functional studies that showed “addiction” of KRAS-mutated cancers to autophagy [91,92,93] suggests that further patient stratification based on molecular alterations is required to fully evaluate the biomarker potential of LC3B in different cancers. For instance, a recent study in non-small cell lung cancer [61] evaluated the punctated pattern of LC3B together with p62 expression levels and pointed to the possibility of improved prognosis in the high-LC3B puncta expression group. It may be informative to further subgroup the patients based on mutation status, including alterations in KRAS.

### 3.2. Other LC3/GABARAP Family Members

Although less studied than LC3B, microtubule-associated protein 1 light chain 3A (LC3A) has also been investigated as an autophagy marker. Earlier studies defined three distinct patterns of IHC staining of LC3A in solid tumors: diffuse cytoplasmic staining, juxtanuclear staining, and staining of “stone-like” structures (SLS) [64]. Each of these patterns appears to bear different prognostic value. For instance, juxtanuclear accumulation of LC3A in tumor cells correlated with good prognosis in colorectal [63] and breast cancers [64], whereas increased numbers of SLS were linked to poor prognosis in colorectal [63], breast [64], gastric [65] and non-small cell lung cancers [66], as well as hepatocellular carcinoma [67] and clear cell ovarian carcinoma [68].

To the best of our knowledge, only one study explored the prognostic significance of another mammalian homolog of yeast Atg8, gamma-aminobutyric acid type A (GABAA) receptor-associated protein (GABARAP), in human cancer. High expression of GABARAP was associated with poor differentiation and shortened overall survival in colorectal cancer patients [69].

### 3.3. p62/SQSTM1

Initially identified as a mediator of NFκB signaling, p62/SQSTM1 is now known as a “signaling hub” for diverse cellular events including amino acid sensing and the oxidative stress response [94]. p62 also functions as a molecular adaptor between the autophagic machinery and its substrates [95]. Due to its degradation during the autophagic process, p62 was proposed to serve as a marker of autophagic flux. For instance, accumulation of p62 protein by Western blot is usually considered indicative of autophagy inhibition [45]. However, caution should be taken when interpreting the results of p62-related assays, as they might be affected by complex regulation of p62 at both the transcriptional and post-translational levels [95]. Despite these known caveats, many authors still use p62 as an indicator of autophagic flux both in vitro and in vivo, and there are numerous publications related to p62 as an autophagy biomarker in human cancer specimens.

With or without a connection to autophagy, the vast majority of reports show an association of high p62 expression with poor prognosis. The examples include, but are not limited to, endometrial cancer, [70], oral squamous cell carcinoma [59], epithelial ovarian cancer [71], and non-small cell lung cancer [72,73], where high expression of cytoplasmic p62 but low expression of nuclear p62 significantly correlated with aggressive tumors and adverse prognosis. In breast cancer, an earlier publication by Rolland et al. [74] showed that p62 cytoplasmic expression correlated with grade, distant metastasis, and reduced five-year survival; in addition, there was a significant association with EGF receptor (EGFR), HER2, HER3, and HER4 expression. High expression of p62 in triple-negative breast cancers was also shown to be prognostic of poor outcome [75]. It would be useful to know whether p62 (both cytoplasmic and nuclear-localized) is differentially expressed and/or has different prognostic values across various molecular subtypes in breast cancer and other cancers. Overall, published data to date indicate that p62 expression is a poor prognostic marker in various cancer types.

### 3.4. ULK-1/2

Being the only serine/threonine kinases in the core autophagy machinery and possibly the most upstream components of the canonical autophagy pathway [7,96,97], ULK-1 and ULK-2 became attractive drug targets [7,8]. While the development of ULK-1/2 small molecule inhibitors is underway [7,8,98], the data on the prognostic value of ULK-1/2 in different cancers is scarce and contradictory.

Tang et al. [80] reported an association of low expression of ULK-1 with adverse patient prognosis in breast cancer. Similarly, ULK-1 was shown to be a favorable prognostic marker in gastric cancer [51]. On the contrary, in colorectal cancer patients, high ULK-1 expression was found to be a predictor of poor prognosis [76]. Another study in colorectal cancer patients [53] did not show any correlation with survival, even after stratification according to KRAS status, but linked ULK-1 high expression to the presence of lymph node metastasis. Poor prognostic value of high ULK-1 expression was also shown in esophageal squamous cell carcinoma [77], hepatocellular carcinoma [78], and nasopharygeal carcinoma [79]. The expression level of ULK-2 was reported to be significantly higher in prostate cancer tissue than in the adjacent normal prostate tissue [99], however no association with prognosis was available from this or other studies. Additional studies in larger patient cohorts are required to better define the prognostic values of ULK1 and ULK2 in different cancer types.

### 3.5. Beclin-1 and VPS34

The Beclin 1-VPS34 complex is one of the central coordinators of autophagy downstream of ULK1 [96]. Several groups reported development of potent VPS34 inhibitors [11,12,13,14,100], but it remains unclear which cancer patients will most likely benefit from these inhibitors.

As Beclin 1 interacts with members of the anti-apoptotic Bcl-2 protein family [101], loss of Beclin 1 expression (allelic loss or suppression by microRNAs) defines poor prognosis presumably by enhancing anti-apoptotic pathways. Overexpression of Beclin 1, linked with tumor hypoxia and acidity, also defines subgroups of tumors with aggressive clinical behavior [82], presumably by promoting autophagy.

Systematic review and meta-analysis by He et al. [48] identified Beclin-1 as a favorable prognostic marker in gastric cancer, breast cancer, lung cancer, and lymphoma, whereas in colorectal cancer the results were split between favorable and poor prognostic values. Another meta-analysis by Han et al. [81] showed that high Beclin-1 expression in patients with colorectal cancer was associated with poor prognosis in terms of tumor distant metastasis and overall survival. Additional studies in colorectal cancer further supported the poor prognostic value of Beclin1 in colorectal cancers [56,82,83]. To address seemingly contradictory reports in colorectal cancer, Han et al. [81] stratified patients according to treatment status and found that high Beclin-1 expression was associated with reduced survival in the patients who received chemotherapy, while among the patients without chemotherapy, high Beclin-1 levels were associated with longer overall survival. Knowing that the majority of chemotherapeutic drugs induce autophagy [40,102], which often plays stress-adaptive roles, we can hypothesize that high Beclin-1 expression in chemotherapy-treated patients indicated increased autophagy levels which promoted resistance to the chemotherapy treatment.

As with other biomarkers in breast cancer, it will be important to examine the expression of Beclin-1 in different histologic and molecular subtypes as was done in a study by Cha et al. [90], who showed that ILC had lower expression of Beclin-1 compared to IDC; in ILC, Beclin-1 expression correlated significantly with ER negativity and was variably expressed according to molecular subtypes, with the highest expression in triple-negative breast cancer.

### 3.6. ATG4B

Due to its central enzymatic roles in the autophagy process, the cysteine protease ATG4B became one of the autophagy proteins being pursued as a potential therapeutic target [9,10], but little is known about its prognostic value in different cancers. Previous reports showed elevated ATG4B expression in colorectal tumor cells [103], chronic myeloid leukemia [104], and lung cancer cells [105]. However, there are no reports in the literature on the prognostic value of ATG4B, or any of the other ATG4 family members, in cancers.

### 3.7. Additional Autophagy-Related Biomarkers

Although not widely pursued or reported in the literature as potential targets, other autophagy-related proteins have been studied in terms of their biomarker potential. The results of these studies are inconsistent and suggest context-dependency. In gastric cancer, low expression of ATG10 and ATG3 were associated with lymph node metastasis and advanced TNM stage; both ATG10 and ATG3 were found to be favorable independent prognostic factors for overall survival [51]. In contrast, the opposite trend was reported in colorectal cancer patients: high ATG10 expression correlated with tumor lymph node metastasis, invasion, and adverse prognosis [84].

Although examined in multiple functional studies involving various in vivo models for autophagy modulation, ATG7 expression did not correlate with survival in lung [106] and gastric [51] cancers. Additional studies are required before eliminating ATG7 as a potential prognostic biomarker.

Similar uncertainty applies to another autophagy-related protein, ATG5. While several studies explored ATG5 expression in different cancers [107,108] only a few reported prognostic values. In breast cancer patients, ATG5, along with FIP200, was shown to be a favorable prognostic marker [85]. In contrast, in oral squamous cell carcinoma, ATG5 expression was associated with high tumor grade, advanced clinical stage, large tumor size, and lymph node metastasis; however, there was no statistically significant correlation with prognosis [109]. Additional studies are also needed to provide a better understanding of biomarker potential of ATG5.

### 3.8. Evaluation of an “Autophagy Signature” in Cancer

Several groups [51,53] have recently attempted to evaluate the expression of multiple autophagy-related proteins simultaneously to determine the prognostic value of a so-called “autophagy signature” in cancer. The idea behind this strategy was that the specific combination of autophagy-related markers evaluated concurrently might reflect the dynamic nature of the autophagy process and its role in tumorigenesis.

Cao et al. [51] assessed prognostic values of 10 markers (ULK1, Beclin 1, ATG3, ATG5, ATG7, ATG9, ATG10, ATG12, LC3B and p62/SQSTM1) in a relatively large cohort of 352 gastric cancer patients. Out of 10 markers, only four (ULK1, Beclin 1, ATG3, and ATG10) demonstrated correlation with prognosis, each being an independent favorable prognostic factor. In the combination analysis, patients with positive expression of all four markers had superior survival compared with those having less than four positive markers [51]. However, no correlation between the markers was reported, which makes the interpretation of these findings difficult. Another study by Ko et al. [86] presented an evaluation of the expression of five autophagy-related proteins—LC3B, ATG5, Beclin 1 and its two cofactors, AMBRA1 (activating molecule in beclin-1-regulated autophagy) and Bif-1 (Bax-interacting factor 1)—in the resected pancreatic ductal adenocarcinoma tissues from a relatively small cohort of 73 patients. The correlation between the expression of autophagy-related proteins was significant for all protein pairs. Multivariate analysis revealed that high beclin-1 expression and high expression of all autophagy-related proteins were independently associated with poor prognosis [86]. The whole cohort was split into “high” and “low” autophagy types according to the number of highly expressed markers (4–5 vs. 0–3, respectively). Although not statistically significant (probably due to the small cohort size), there was a trend towards decreased overall survival in the “high autophagy type” group [86]. In most other studies, though, the so-called “autophagy signature” included 2–4 markers only [50,53,56,85,89,109], often making the results difficult to interpret.

### 3.9. Secreted Factors as Potential Predictive Biomarkers

In addition to lysosomal degradation of autophagosomal contents, a novel role for autophagy that involves unconventional protein secretion was described [110]. Another recent study by Kraya et al. [111], which utilized quantitative proteomics to identify secreted factors characteristic of tumor cells with high autophagy levels compared to low-autophagy tumor cells, opened up an exciting possibility of indirect measurement of tumoral autophagy dynamics in plasma. Unique secreted factors may serve as predictive biomarkers and aid in patient selection for treatment involving autophagy inhibition, and might also be useful for patient follow-up to evaluate the subsequent response to a given treatment. These factors included LIF (leukemia inhibitory factor), IL1B (interleukin 1, β), CXCL8 (chemokine (C-X-C motif) ligand 8), FAM3C (family with sequence similarity 3, member C), and DKK3 (dickkopf WNT signaling pathway inhibitor 3)—factors implicated in immunity and inflammation [111]. The authors chose melanoma as a model, and although this particular set of markers validated well in both in vitro and in vivo (patient specimens), further studies are needed to show whether these candidate biomarkers are relevant for other cancer subtypes or whether the autophagy-dependent secretome signatures are context (i.e., cancer type)-specific. Nonetheless, with growing evidence for the role of autophagy in immunity, inflammation, and the tumor microenvironment [112,113,114], as well as the promises of “liquid biopsies”, great attention should be given to the development in this direction.

## 4. Discussion and Future Directions

In our previous review in 2012 [32] we summarized the literature regarding expression levels of autophagy proteins in various cancer tissues. At that time, no clear picture could be drawn from the published data, and there existed many examples of inconsistent and conflicting reports on autophagy protein expression patterns. The reasons for these discrepancies included small patient cohorts, absence of standard techniques to process the tissues and analyze the results, lack of independent validation cohorts, and a limited number of autophagy-related markers (LC3, p62, and Beclin-1) available for assessment in patient samples.

In this review, we highlight the more recent advances in the evaluation of the biomarker potential of autophagy proteins in various cancers. Over the past five years, multiple groups accumulated data regarding IHC assessment of additional autophagy-related biomarkers in human cancer specimens (Table 1). While patterns have emerged (Table 1), these studies also showed that prognostic values of specific autophagy-related proteins, some of which are currently pursued as therapeutic targets, vary from one tumor type to another, from one disease stage to another, and between untreated and treated patients. These findings further support the context-dependent roles of autophagy in cancer, and also emphasize the need to interpret biomarker data in a corresponding context-dependent manner. Appreciation of the complexity and dynamic nature of the autophagy process has translated to increased interest in the evaluation of so-called “autophagy signatures” in different malignancies. This approach, focused on simultaneous assessment of multiple proteins in the autophagy pathway, deserves further attention along with the investigation of autophagy associated secreted factors.

Across the large and growing number of autophagy-related biomarker studies, the main challenges encountered previously still remain for the most part unresolved. These challenges include differences in staining techniques, scoring systems and cut-off points, limited sample sizes, and absence of independent validation processes. In addition, it is important to emphasize that knowing the prognostic relevance of autophagy-related proteins does not necessarily translate into their predictive value for the associated targeted therapeutics. New information regarding autophagy-related predictive biomarkers from the ongoing or future clinical trials is highly anticipated.

The question of how to track response to autophagy inhibitors in cancer patients remains one of the most outstanding in the field. An increase or decrease in the expression level of autophagosomal markers (such as LC3B) may not directly correlate with changes in autophagic activity, and is difficult to interpret, as well as monitor over time, especially in tissues. A recent attempt by Mahalingam et al. [115] to analyze peripheral blood mononuclear cells (PBMCs) from patients treated with HCQ and vorinostat suggested that tumor samples, although not readily accessible, are far more informative for the assessment of autophagy inhibition in vivo compared to PBMCs. In this study, in addition to LC3B and p62 levels, the authors evaluated the levels of the lysosomal protease cathepsin D (CTSD), which they previously showed to be a key mediator of CQ/HCQ and HCQ plus vorinostat-induced apoptosis. In this context, novel biomarkers indirectly related to autophagic activity and its modulation, such as autophagy products rather than autophagy machinery, should also be explored.

Autophagy is a highly regulated process, and autophagy proteins are subject to regulatory post-translational modifications, including phosphorylation, ubiquitination, and acetylation [116]. It is possible, although technically challenging, that the evaluation of the expression of modified (e.g., phosphorylated) forms of autophagy-related proteins in tumor tissues, which might be feasible via mass spectrometry-based approaches [117], will provide useful information regarding the biomarker potential of these proteins in cancers, and this is an important area for future development.

Although this review focuses on IHC evaluation of autophagy-related protein levels in cancer tissues, it should be noted that additional techniques have been considered for the discovery and monitoring of autophagy-related alterations and their biomarker potential in various cancers. For instance, Rothe et al. [104] examined transcript levels of several key autophagy and autophagy-related genes in chronic myeloid leukemia (CML) stem cells, and showed that ATG4B and ATG5 were differentially expressed in imatinib-nonresponders vs. responders. The authors of this study suggested that the unique autophagy gene expression signature may serve as a novel, clinically useful biomarker for predicting response to tyrosine kinase inhibitor therapy. Eissa et al. [118] identified and validated a novel autophagy transcript signature for the diagnosis of human bladder cancer. In this study, the expression levels of a number of autophagy genes, including ATG12 and ULK1, in paired bladder tissue and urine samples were significantly lower in bladder cancer than in the control group. A large-scale analysis of the mutational status of the genes encoding the entire core autophagy machinery, published by Lebovitz et al. [119], indicated that the core autophagy machinery largely escapes genomic alterations in human cancers. However, those tumors in which mutations were found, should be given further attention [93].

Overall, significant progress has been made over the last several years, and the development of new strategies and standardized approaches for the comprehensive evaluation of the multiplayer and multistep autophagy process in human tissues is underway.

## Figures and Tables

**Table 1:** Prognostic values of autophagy-related proteins in cancers.

Candidate Marker *	Prognostic Value **	
	Poor	Favorable
LC3B	BC [22,48,49], GC [50,51,52], CRC [53,54,55,56], melanoma [22], astrocytoma [57], esophageal cancer [58], oral SCC [59], and HCC [60]	NSCLC [61], BC [62]
LC3A	CRC [63], BC [64], GC [65], NSCLCs [66], HCC [67] and clear cell OC [68] (stone-like structures)	CRC [63] and BC [64] (juxtanuclear accumulation)
GABARAP	CRC patients [69]	
p62	endometrial cancer, [70], oral SCC [59], epithelial OC [71], and NSCLC [72,73], BC [74,75]	
ULK1	CRC [76], esophageal SCC [77], HCC [78], and nasopharygeal carcinoma [79]	BC [80], GC [51]
Beclin1	CRC [48,81] ***; [56,82,83]	GC, BC, NSCLC, CRC, lymphoma [48] ***
ATG3		GC [51]
ATG10	CRC [84]	GC [51]
FIP200		BC [85]
Autophagy “signature”: ULK1, Beclin 1, ATG3, and ATG10		GC [51]
Autophagy “signature”: LC3B, ATG5, Beclin 1, Ambra1 and Bif-1	PDAC [86]	

* Protein expression was evaluated by immunohistochemistry; ** Prognostic value was based on the association of high protein expression with patient outcomes. BC, breast cancer; GC, gastric cancer; CRC, colorectal cancer; HCC, hepatocellular carcinoma; NSCLC, non-small cell lung cancer; PDAC, pancreatic ductal adenocarcinoma; OC, ovarian cancer; SCC, squamous cell carcinoma; *** based on meta-analysis of multiple publications.
